# Supplementary material for: Gram staining decipherment using an artificial intelligence-powered smartphone-based application
Source: Microbiol Spectr. 2026 Apr 27;14(6):e03076-25. doi: 10.1128/spectrum.03076-25 (PMC13228010; doi:10.1128/spectrum.03076-25)
Supplement: Supplemental material — Text S1 to S3, Tables S1 to S3, and Fig. S1 and S2. [file spectrum.03076-25-s0001.docx]

**Supplemental material**

Text S1. Artificial intelligence (AI) model building

*Data collection*

1. The 1,350 specimens for AI model building were collected between April 2020 and December 2023 (excluding between April 2022 and December 2022), from existing anonymized National Center for Global Health and Medicine (NCGM) and Kobe University Hospital (KUH) registries.
2. The bacterial species and the number of slides collected.

|  | **Slides from NCGM** | **Slides from KUH** | **Total** |
| --- | --- | --- | --- |
| Polymicrobial | 144 | 18 | 162 |
| None | 46 | 14 | 60 |
| *Candida* spp. | 103 | 33 | 136 |
| GPC cluster | 63 | 22 | 85 |
| *Enterococcus faecalis* | 37 | 30 | 67 |
| *Enterococcus faecium* | 19 | 20 | 39 |
| *Streptococcus agalactiae* | 22 | 21 | 43 |
| Other GPC | 15 | 20 | 35 |
| *Corynebacterium* spp. | 32 | 38 | 70 |
| *Enterobacter cloacae* | 16 | 27 | 43 |
| *Escherichia coli* | 235 | 56 | 291 |
| *Klebsiella oxytoca* | 18 | 28 | 46 |
| *Klebsiella pneumoniae* | 65 | 27 | 92 |
| Other GNR *Enterobacteriaceae* | 65 | 20 | 85 |
| *Pseudomonas aeruginosa* | 32 | 15 | 47 |
| Other GNR glucose non-fermenting bacteria | 5 | 22 | 27 |
| GNC | 7 | 10 | 17 |
| *Others | 5 | - | 5 |
| Total | 929 | 421 | 1350 |
| NCGM, National Centre for Global Health and Medicine; KUH, Kobe University Hospital; GPC, gram-positive cocci; GNC, gram-negative cocci; GNR, gram-negative rod  * Unlike the general definition of polymicrobial samples, if two different bacterial types were observed within the GNR category or within the GPC category on the same slide, these slides were included for training as Class 1 (one GNR slide and four GPC slides), but they were not used for Class 2 classification | | | |

*AI model building*

1. We used Python as the language and PyTorch (https://pytorch.org/) along with its wrapper PyTorch Lightning (https://lightning.ai/pytorch-lightning) as the deep learning framework to construct the AI model.
2. A total of 13,901 images (from 1,350 slides) were used to construct the AI model.
3. Data splitting was performed on a per-slide basis, rather than on the entire image, to prevent the learning of slide-by-slide features rather than bacterial features. This ensured that the images derived from the same slide did not cross over to the training, validation, or testing datasets.
4. We created three types of models: a model that classified seven categories of morphology, including 1) yeast (*Candida* spp.), 2) gram-positive cocci (GPC), 3) gram-positive rods (GPR), 4) gram-negative rods (GNR), 5) gram-negative cocci (GNC), 6) nonfermenting bacterial classes, and 7) multiple bacterial classes (Class 1 model). There was one model that classified bacterial species as GPC in the Class 1 model (GPC model), and another that classified them as GNR in the Class 1 model (GNR model).
5. We created the Class 1 model as a multi-label classification model, and the GPC and GNR models as multi-class image classification models.
6. We used a fine tuning technique based on the ConvNeXt (https://doi.org/10.1109/CVPR52688.2022.01167) convolutional neural network (CNN) architecture pretrained on the ImageNet Large Scale Visual Recognition Competition (ILSVRC) 2012 image database (https://doi.org/10.1007/s11263-015-0816-y).
7. We used 20% of all the slides as the test dataset and divided the remaining 80% into four parts. We performed four-fold cross-validation (4-fold CV) using three of the four parts as the training dataset (training) and the remaining part as the validation dataset. We then built a model. As there was only one clinical specimen for GNC, the images of the clinical specimens were divided in the same proportion to construct the dataset.
8. The batch size was set to 32 because we used gradient accumulation with minibatch sizes of 2 and 16.
9. For the loss function, we used multi-label evidential loss (https://doi.org/10.1109/ICASSP49357.2023.10096569) for the Class 1 model and evidential loss (https://dl.acm.org/doi/pdf/10.5555/3327144.3327239) for the remaining two models.
10. We used AdamW (https://openreview.net/pdf?id=Bkg6RiCqY7) as the optimizer.
11. Data augmentation was performed using the RandAugment (https://doi.org/10.1109/CVPRW50498.2020.00359) module in PyTorch.
12. To construct the Class 1 model, we used an augmentation method called mixup (https://openreview.net/pdf?id=r1Ddp1-Rb), which combines multiple images.
13. As evaluation metrics, we used accuracy, recall, precision, and f_β (β=0.5). We monitored the recall of the validation data and selected the model that achieved the highest score. The f_β score was based on the following equation:

$$f_{\beta}=\left( 1+\beta^{2} \right)\frac{precision\cdot recall}{\beta^{2}\cdot precision+recall}$$

14. We built a model by optimizing the weights using the Model Soups (https://proceedings.mlr.press/v162/wortsman22a/wortsman22a.pdf) method on the models constructed using the 4-fold CV.

*Model evaluation*

1. During inference, the image was resized to 1024 × 1024 pixels after center cropping to 3024 × 3024 pixels and inputted into the Class 1 model.
2. If the output of the Class 1 model was GNR or GPC, the corresponding model was used to classify the fungal species, and the Class 1 category and fungal classification results were the output.
3. If evidential loss was used as the loss function, the uncertainty u and confidence conf were calculated as follows:

$$f\left( x \right)=\left\{ \begin{aligned} x \left( x>0 \right) \\ 0 \left( x\leq0 \right) \end{aligned} \right.$$

$$u=\frac{K}{\sum_{k=0}^{K} \left( f_{k}\left( x \right)+1 \right)}$$

$$conf=1-u$$

where K is the number of classification classes.

1. When multi-label evidential loss was used as the loss function, the uncertainty was calculated for each class according to the following equation using two outputs: present (+) and absent (−) for each class.

$$u_{k}=\frac{2}{\left( f_{k}^{+}\left( x \right)+1 \right)+ \left( f_{k}^{-}\left( x \right)+1 \right)}$$

The maximum value was the output from the following equation as the uncertainty of the image:

$$u=max\left( \left\{ u_{k} \right\}_{k=1}^{K} \right)$$

1. In addition to confidence, the probabilities were calculated using a sigmoid function for the Class 1 model and a softmax function for the GNR and GPC models.

Text S2. Procedure for preparing the spiked specimens using American Type Culture Collection (ATCC) standard strains

1. The stored strain was incubated on CO_2_-rich chocolate agar medium for 24 h.
2. The isolated strain was adjusted to a concentration of McFarland 0.5, and further diluted 100-fold.
3. We inoculated 3 mL of medium supplemented with cation-adjusted Muller-Hinton Bouillon + horse hemolytic blood 2.5–5% with 10 µL of the adjusted solution in (2).
4. After incubation in CO_2_ rich medium overnight (approximately 16–18 h), the culture medium was suspended in bacteria-free urine or saline solution of the same pH as the urine and applied on a glass slide for Gram staining.

American Type Culture Collection (ATCC) strains used in this study:

Kobe university hospital:

*Enterococcus faecium* ATCC27270

*Enterococcus faecium* ATCC35667

*Aerococcus viridans* ATCC10400

*Klebsiella oxytoca* ATCC49131

*Klebsiella oxytoca* ATCC8724

*Alcaligenes faecalis* ATCC8750

*Stenotrophomonas maltophilia* ATCC17666

*Stenotrophomonas maltophilia* ATCC51331

*Neisseria gonorrhoeae* ATCC19424

*Neisseria meningitidis* ATCC13077

*Neisseria gonorrhoeae* ATCC31426

Natinal Center for Global Health and Medicine:

*Acinetobacter lwoffii* ATCC17925

*Acinetobacter* species ATCC49139

*Stenotrophomonas maltophilia* ATCC13637

*Neisseria gonorrhoeae* ATCC31426

*Neisseria gonorrhoeae* ATCC19424

*Neisseria gonorrhoeae* ATCC43070

Text S3. Decipherment

For the computer-aided diagnostic system (CAD) used in this study, deep learning with supervised data generated from gram-stained slides was collected from KUH and NCGM outside the study period and registered. The image datasets for decipherment created at the two facilities were sent to different facilities in an anonymized form. Ten microbiology specialists (MS) from each facility performed the decipherment on a PC display. After MS deciphering was completed, the same image dataset was sent to the CarbGeM laboratory for deciphering using CAD. After the CAD deciphering was completed, both decipherment results were sent to each facility where the dataset was created, and the results were checked against the correct data.

**TABLE S1** Eligible samples

|  | **NCGM** | **KUH** |
| --- | --- | --- |
| *Candida* spp. | 29 | 13 |
| GPC cluster | 31 | 18 |
| *Enterococcus faecalis* | 18 | 9 |
| *Enterococcus faecium* | 5 | 1 |
| *Streptococcus agalactiae* | 11 | 3 |
| Other GPC | 4 | 2 |
| *Corynebacterium* spp. | 14 | 5 |
| *Enterobacter cloacae* | 6 | 3 |
| *Escherichia coli* | 87 | 26 |
| *Klebsiella oxytoca* | 7 | 1 |
| *Klebsiella pneumoniae* | 17 | 13 |
| Other GNR *Enterobacteriaceae* | 13 | 11 |
| *Pseudomonas aeruginosa* | 16 | 5 |
| Other GNR glucose non-fermenting bacteria | 0 | 0 |
| GNC | 0 | 0 |
| Polymicrobial | 292 | 419 |
| None | 838 | 1,340 |
| Total | 1,388 | 1,869 |

GPC, gram-positive cocci; GNC, gram-negative cocci; GNR, gram-negative rods

**TABLE S2** Performance of the computer-aided diagnostic system for Class 2

1. Dataset 1 (by device)

|  | **All** | **Microscopic camera** | **Galaxy** | **iPhone** | **AQUOS** | **Xperia** |
| --- | --- | --- | --- | --- | --- | --- |
| Accuracy | 0.64 | 0.68 | 0.64 | 0.64 | 0.61 | 0.63 |
| 95% CI | 0.63 to 0.65 | 0.65 to 0.70 | 0.62 to 0.67 | 0.62 to 0.66 | 0.58 to 0.63 | 0.61 to 0.66 |
| Kappa | 0.55 | 0.60 | 0.55 | 0.55 | 0.51 | 0.54 |
| Macro recall | 0.38 | 0.43 | 0.36 | 0.36 | 0.36 | 0.40 |
| Balanced accuracy | | | | | | |
| Polymicrobial | 0.82 | 0.86 | 0.84 | 0.84 | 0.77 | 0.80 |
| None | 0.94 | 0.95 | 0.94 | 0.94 | 0.92 | 0.95 |
| U-01 | 0.99 | 1.00 | 1.00 | 0.96 | 0.99 | 1.00 |
| U-02 | 0.86 | 0.89 | 0.87 | 0.83 | 0.84 | 0.86 |
| U-03 | 0.69 | 0.70 | 0.65 | 0.64 | 0.73 | 0.73 |
| U-04 | 0.58 | 0.61 | 0.54 | 0.61 | 0.53 | 0.58 |
| U-05 | 0.70 | 0.76 | 0.64 | 0.66 | 0.71 | 0.74 |
| U-06 | 0.55 | 0.60 | 0.51 | 0.51 | 0.58 | 0.57 |
| U-07 | 0.85 | 0.89 | 0.86 | 0.82 | 0.79 | 0.87 |
| U-08 | 0.51 | 0.51 | 0.49 | 0.49 | 0.53 | 0.51 |
| U-09 | 0.81 | 0.84 | 0.78 | 0.85 | 0.71 | 0.85 |
| U-10 | 0.50 | 0.50 | 0.49 | 0.49 | 0.51 | 0.51 |
| U-11 | 0.53 | 0.52 | 0.51 | 0.56 | 0.55 | 0.51 |
| U-12 | 0.54 | 0.67 | 0.54 | 0.50 | 0.50 | 0.50 |
| U-13 | 0.67 | 0.71 | 0.72 | 0.61 | 0.66 | 0.66 |
| U-14 | 0.51 | 0.50 | 0.50 | 0.54 | 0.50 | 0.51 |
| U-15 | 0.51 | 0.50 | 0.50 | 0.50 | 0.51 | 0.51 |
| F1 value | | | | | | |
| Polymicrobial | 0.77 | 0.83 | 0.80 | 0.79 | 0.70 | 0.75 |
| None | 0.90 | 0.92 | 0.90 | 0.91 | 0.86 | 0.90 |
| U-01 | 0.98 | 0.99 | 1.00 | 0.94 | 0.98 | 0.98 |
| U-02 | 0.64 | 0.60 | 0.65 | 0.65 | 0.61 | 0.69 |
| U-03 | 0.32 | 0.33 | 0.28 | 0.27 | 0.39 | 0.35 |
| U-04 | 0.26 | 0.36 | 0.16 | 0.36 | 0.11 | 0.27 |
| U-05 | 0.37 | 0.41 | 0.28 | 0.34 | 0.38 | 0.43 |
| U-06 | 0.18 | 0.30 | 0.05 | 0.05 | 0.23 | 0.22 |
|  | All | Microscopic camera | Galaxy | iPhone | AQUOS | Xperia |
| U-07 | 0.49 | 0.50 | 0.48 | 0.50 | 0.44 | 0.50 |
| U-08 | 0.04 | 0.05 | NA | NA | 0.09 | 0.04 |
| U-09 | 0.25 | 0.31 | 0.25 | 0.26 | 0.22 | 0.23 |
| U-10 | 0.02 | NA | NA | NA | 0.04 | 0.05 |
| U-11 | 0.10 | 0.08 | 0.04 | 0.18 | 0.14 | 0.04 |
| U-12 | 0.12 | 0.28 | 0.11 | NA | 0.03 | NA |
| U-13 | 0.30 | 0.44 | 0.35 | 0.21 | 0.25 | 0.28 |
| U-14 | 0.04 | NA | NA | 0.14 | NA | 0.05 |
| U-15 | 0.02 | NA | NA | NA | 0.05 | 0.05 |
| Precision | | | | | | |
| Polymicrobial | 0.92 | 0.95 | 0.90 | 0.87 | 0.94 | 0.97 |
| None | 0.84 | 0.88 | 0.85 | 0.87 | 0.76 | 0.84 |
| U-01 | 0.97 | 0.98 | 1.00 | 0.95 | 0.98 | 0.95 |
| U-02 | 0.56 | 0.48 | 0.57 | 0.63 | 0.54 | 0.66 |
| U-03 | 0.27 | 0.26 | 0.23 | 0.23 | 0.32 | 0.27 |
| U-04 | 0.82 | 0.89 | 1.00 | 0.89 | 1.00 | 0.60 |
| U-05 | 0.33 | 0.32 | 0.26 | 0.36 | 0.32 | 0.37 |
| U-06 | 0.51 | 0.70 | 0.50 | 0.50 | 0.38 | 0.56 |
| U-07 | 0.37 | 0.36 | 0.35 | 0.40 | 0.35 | 0.37 |
| U-08 | 0.05 | 0.11 | 0.00 | 0.00 | 0.10 | 0.09 |
| U-09 | 0.15 | 0.20 | 0.15 | 0.16 | 0.14 | 0.13 |
| U-10 | 0.02 | 0.00 | 0.00 | 0.00 | 0.08 | 0.14 |
| U-11 | 0.23 | 0.29 | 0.17 | 0.29 | 0.21 | 0.14 |
| U-12 | 0.14 | 0.22 | 0.12 | 0.00 | 0.04 | 0.00 |
| U-13 | 0.25 | 0.44 | 0.28 | 0.19 | 0.18 | 0.24 |
| U-14 | 0.20 | NA | 0.00 | 0.43 | 0.00 | 1.00 |
| U-15 | 0.67 | NA | NA | NA | 0.50 | 1.00 |
| Recall | | | | | | |
| Polymicrobial | 0.67 | 0.73 | 0.72 | 0.73 | 0.56 | 0.61 |
| None | 0.97 | 0.96 | 0.96 | 0.95 | 0.99 | 0.98 |
| U-01 | 0.98 | 1.00 | 1.00 | 0.93 | 0.98 | 1.00 |
| U-02 | 0.73 | 0.80 | 0.75 | 0.68 | 0.70 | 0.73 |
| U-03 | 0.41 | 0.44 | 0.33 | 0.31 | 0.49 | 0.49 |
|  | All | Microscopic camera | Galaxy | iPhone | AQUOS | Xperia |
| U-04 | 0.15 | 0.23 | 0.09 | 0.23 | 0.06 | 0.17 |
| U-05 | 0.43 | 0.55 | 0.30 | 0.33 | 0.45 | 0.50 |
| U-06 | 0.11 | 0.19 | 0.03 | 0.03 | 0.17 | 0.14 |
| U-07 | 0.73 | 0.83 | 0.75 | 0.68 | 0.60 | 0.78 |
| U-08 | 0.03 | 0.03 | 0.00 | 0.00 | 0.09 | 0.03 |
| U-09 | 0.72 | 0.75 | 0.65 | 0.83 | 0.50 | 0.85 |
| U-10 | 0.01 | 0.00 | 0.00 | 0.00 | 0.03 | 0.03 |
| U-11 | 0.07 | 0.04 | 0.02 | 0.13 | 0.11 | 0.02 |
| U-12 | 0.10 | 0.38 | 0.10 | 0.00 | 0.03 | 0.00 |
| U-13 | 0.38 | 0.44 | 0.47 | 0.24 | 0.38 | 0.36 |
| U-14 | 0.02 | 0.00 | 0.00 | 0.08 | 0.00 | 0.03 |
| U-15 | 0.01 | 0.00 | 0.00 | 0.00 | 0.03 | 0.03 |

1. Dataset 2 (by staining method)

|  | **All** | **B&M** | **Favor** |
| --- | --- | --- | --- |
| Accuracy | 0.67 | 0.69 | 0.64 |
| 95% CI | 0.65 to 0.69 | 0.66 to 0.72 | 0.62 to 0.67 |
| Kappa | 0.59 | 0.62 | 0.57 |
| Macro recall | 0.47 | 0.50 | 0.44 |
| Balanced accuracy | | | |
| Polymicrobial | 0.85 | 0.83 | 0.87 |
| None | 0.96 | 0.98 | 0.94 |
| U-01 | 0.97 | 0.97 | 0.97 |
| U-02 | 0.76 | 0.77 | 0.75 |
| U-03 | 0.85 | 0.89 | 0.80 |
| U-04 | 0.50 | 0.50 | 0.50 |
| U-05 | 0.64 | 0.63 | 0.66 |
| U-06 | 0.60 | 0.63 | 0.56 |
| U-07 | 0.93 | 0.91 | 0.96 |
| U-08 | 0.52 | 0.55 | 0.50 |
| U-09 | 0.74 | 0.69 | 0.78 |
| U-10 | 0.75 | 0.79 | 0.70 |
| U-11 | 0.55 | 0.57 | 0.53 |
|  | All | B&M | Favor |
| U-12 | 0.53 | 0.52 | 0.54 |
| U-13 | 0.82 | 0.78 | 0.87 |
| U-14 | 0.59 | 0.61 | 0.57 |
| U-15 | 0.76 | 1.00 | 0.51 |
| F1 value | | | |
| Polymicrobial | 0.77 | 0.76 | 0.78 |
| None | 0.95 | 0.97 | 0.93 |
| U-01 | 0.80 | 0.80 | 0.79 |
| U-02 | 0.57 | 0.64 | 0.51 |
| U-03 | 0.52 | 0.50 | 0.54 |
| U-04 | 0.02 | NA | 0.03 |
| U-05 | 0.33 | 0.27 | 0.41 |
| U-06 | 0.27 | 0.35 | 0.18 |
| U-07 | 0.70 | 0.74 | 0.66 |
| U-08 | 0.09 | 0.15 | NA |
| U-09 | 0.33 | 0.28 | 0.38 |
| U-10 | 0.35 | 0.49 | 0.26 |
| U-11 | 0.16 | 0.19 | 0.12 |
| U-12 | 0.10 | 0.07 | 0.13 |
| U-13 | 0.48 | 0.43 | 0.52 |
| U-14 | 0.27 | 0.31 | 0.22 |
| U-15 | 0.68 | 1.00 | 0.05 |
| Precision | | | |
| Polymicrobial | 0.81 | 0.85 | 0.78 |
| None | 0.96 | 0.98 | 0.95 |
| U-01 | 0.68 | 0.69 | 0.68 |
| U-02 | 0.61 | 0.76 | 0.50 |
| U-03 | 0.40 | 0.35 | 0.48 |
| U-04 | 0.04 | 0.00 | 0.05 |
| U-05 | 0.36 | 0.26 | 0.55 |
| U-06 | 0.38 | 0.48 | 0.26 |
| U-07 | 0.57 | 0.67 | 0.51 |
| U-08 | 0.18 | 0.25 | 0.00 |
|  | All | B&M | Favor |
| U-09 | 0.23 | 0.20 | 0.27 |
| U-10 | 0.26 | 0.41 | 0.18 |
| U-11 | 0.27 | 0.25 | 0.33 |
| U-12 | 0.15 | 0.10 | 0.20 |
| U-13 | 0.37 | 0.34 | 0.39 |
| U-14 | 0.52 | 0.50 | 0.56 |
| U-15 | 1.00 | 1.00 | 1.00 |
| Recall | | | |
| Polymicrobial | 0.73 | 0.69 | 0.78 |
| None | 0.94 | 0.97 | 0.91 |
| U-01 | 0.95 | 0.95 | 0.95 |
| U-02 | 0.53 | 0.56 | 0.51 |
| U-03 | 0.73 | 0.83 | 0.63 |
| U-04 | 0.01 | 0.00 | 0.03 |
| U-05 | 0.31 | 0.28 | 0.33 |
| U-06 | 0.21 | 0.28 | 0.14 |
| U-07 | 0.89 | 0.83 | 0.94 |
| U-08 | 0.06 | 0.11 | 0.00 |
| U-09 | 0.54 | 0.45 | 0.63 |
| U-10 | 0.54 | 0.61 | 0.47 |
| U-11 | 0.11 | 0.15 | 0.08 |
| U-12 | 0.08 | 0.05 | 0.10 |
| U-13 | 0.69 | 0.60 | 0.77 |
| U-14 | 0.18 | 0.22 | 0.14 |
| U-15 | 0.51 | 1.00 | 0.03 |

CI, confidence interval

**TABLE S3** Agreement between computer-aided diagnostic system predictions by device and staining method

1. Devices (Dataset 1) for Class 1

|  | **Microscopic camera** | **Galaxy** | **iPhone** | **AQUOS** | **Xperia** | |
| --- | --- | --- | --- | --- | --- | --- |
| Accuracy | 84% | 81% | 81% | 76% | 80% | |
| Correct | 1331 | 1284 | 1284 | 1213 | 1270 | |
| Incorrect | 257 | 304 | 304 | 375 | 318 | |
| *p* value for pairwise comparisons | | | | | |
| Microscopic camera | - | 0.046 | 0.046 | <0.001 | 0.014 | |
| Galaxy | - | - | 1.0 | 0.008 | 0.62 | |
| iPhone | - | - | - | 0.008 | 0.62 | |
| AQUOS | - | - | - | - | 0.032 | |

1. Devices (Dataset 1) for Class 2

|  | **Microscopic camera** | **Galaxy** | **iPhone** | **AQUOS** | **Xperia** |
| --- | --- | --- | --- | --- | --- |
| Accuracy | 68% | 64% | 64% | 61% | 63% |
| Correct | 1076 | 1022 | 1017 | 962 | 1002 |
| Incorrect | 512 | 566 | 571 | 626 | 586 |
| *p* value for pairwise comparisons | | | | | |
| Microscopic camera | - | 0.080 | 0.076 | <0.001 | 0.032 |
| Galaxy | - | - | 0.88 | 0.076 | 0.60 |
| iPhone | - | - | - | 0.080 | 0.67 |
| AQUOS | - | - | - | - | 0.22 |

1. Staining method (Dataset 2) for Class 1

|  | **B&M** | **Favor** |
| --- | --- | --- |
| Accuracy | 91% | 86% |
| Correct | 1079 | 1014 |
| Incorrect | 103 | 168 |
| *p* value for Fisher’s exact test: <0.001 | | |

1. Staining method (Dataset 2) for Class 2

|  | **B&M** | **Favor** |
| --- | --- | --- |
| Accuracy | 69% | 64% |
| Correct | 815 | 760 |
| Incorrect | 367 | 422 |
| *p* value for Fisher’s exact test: 0.018 | | |

B&M, Bartholomew and Mittwer

| 1. Dataset 1, Class 1 | d) Dataset 1, Class 2 |
| --- | --- |
| 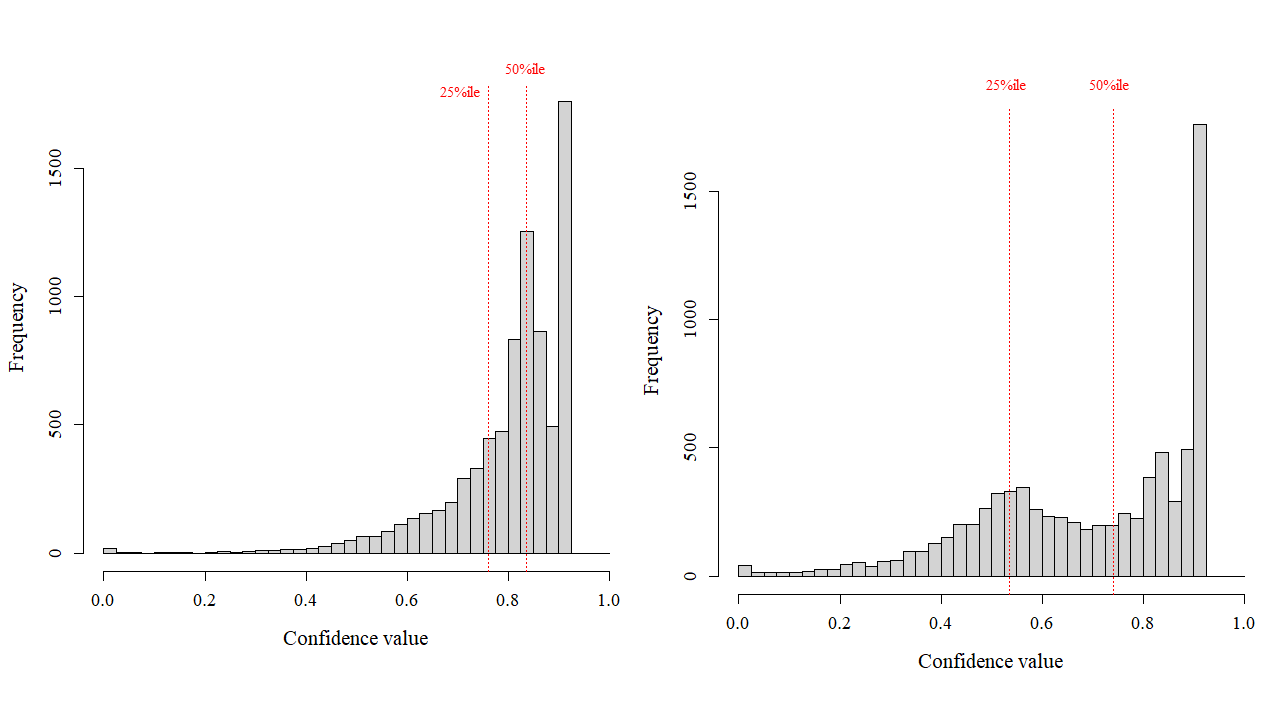 | 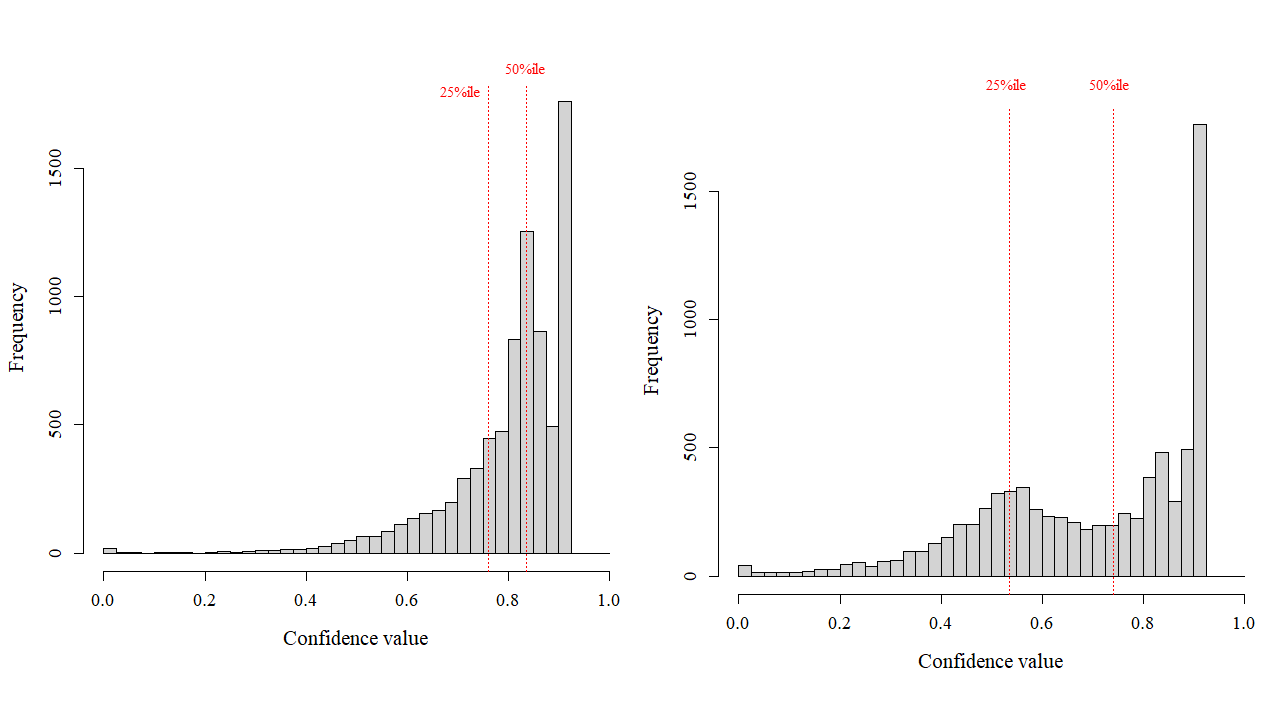 |

| 1. Dataset 2, Class 1 | 1. Dataset 2, Class 2 |
| --- | --- |
| 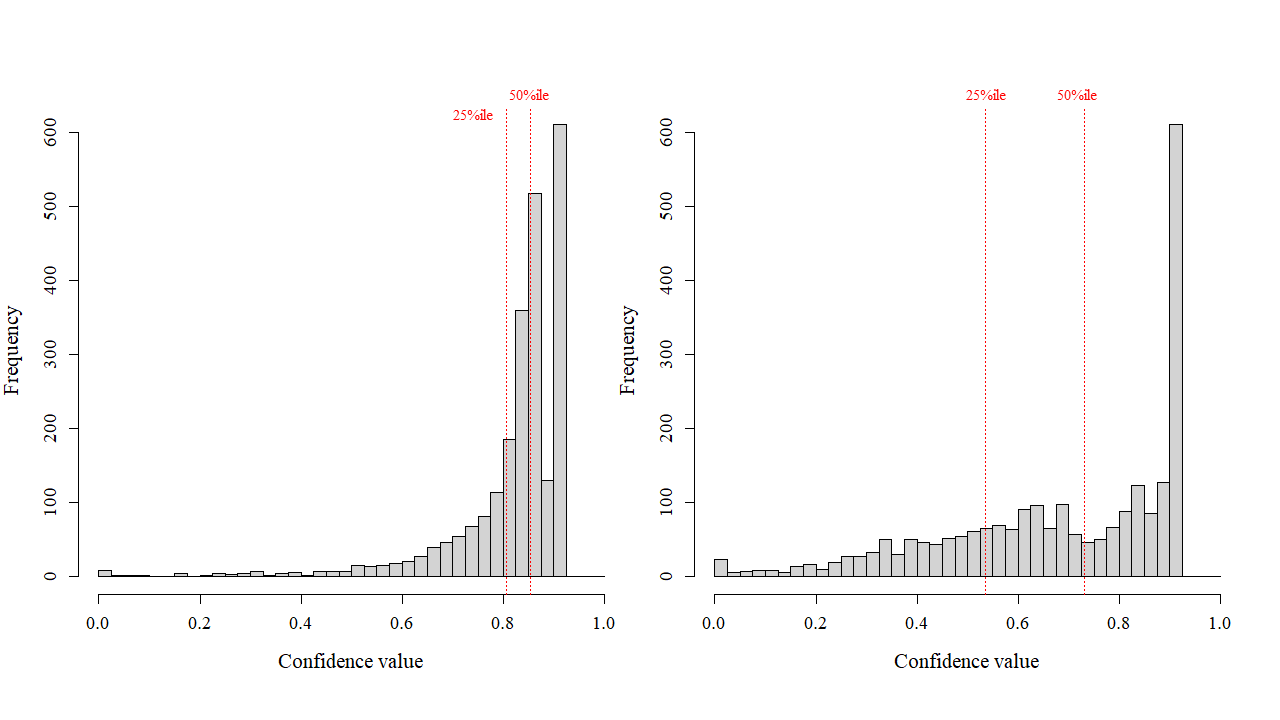 | 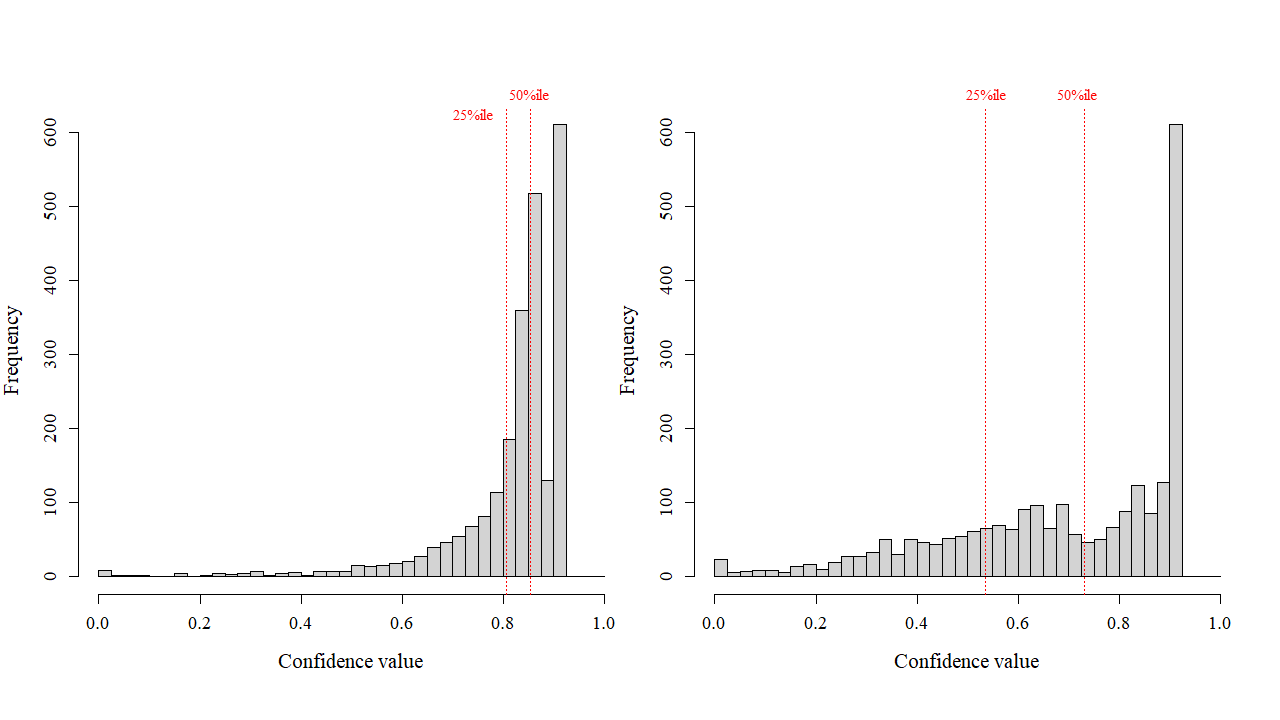 |

Fig. S1. Distribution of the confidence values

| 1. Dataset 1, Class 1 | 1. Dataset 1, Class 2 |
| --- | --- |
| 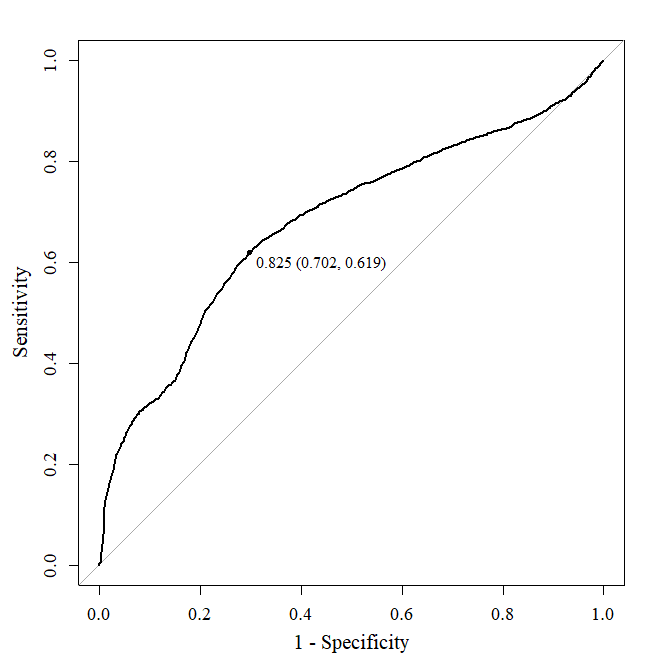 | 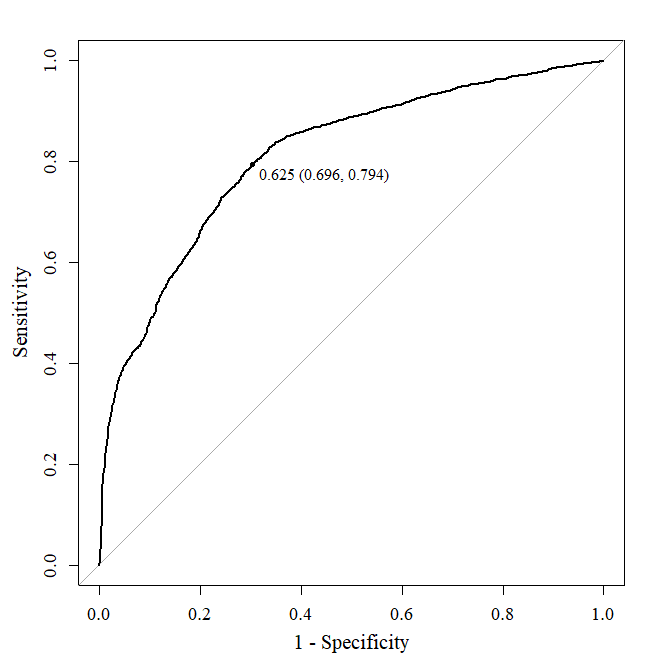 |

| 1. Dataset 2, Class 1 | 1. Dataset 2, Class 2 |
| --- | --- |
| 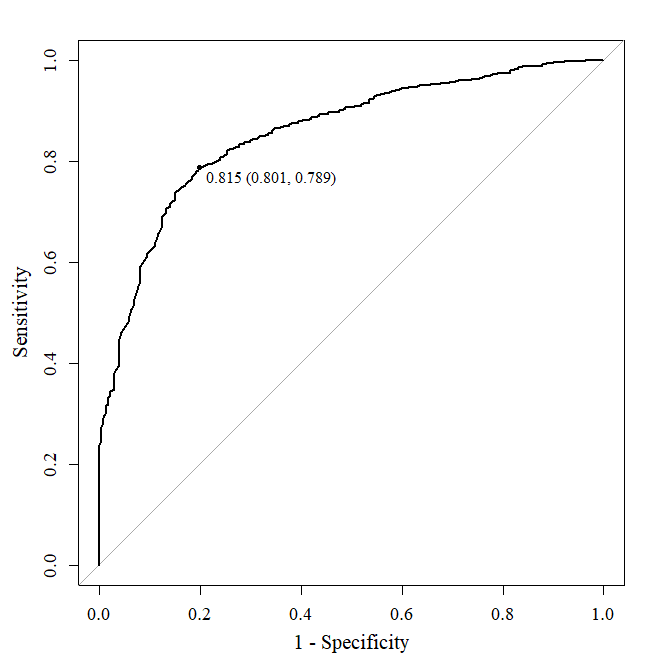 | 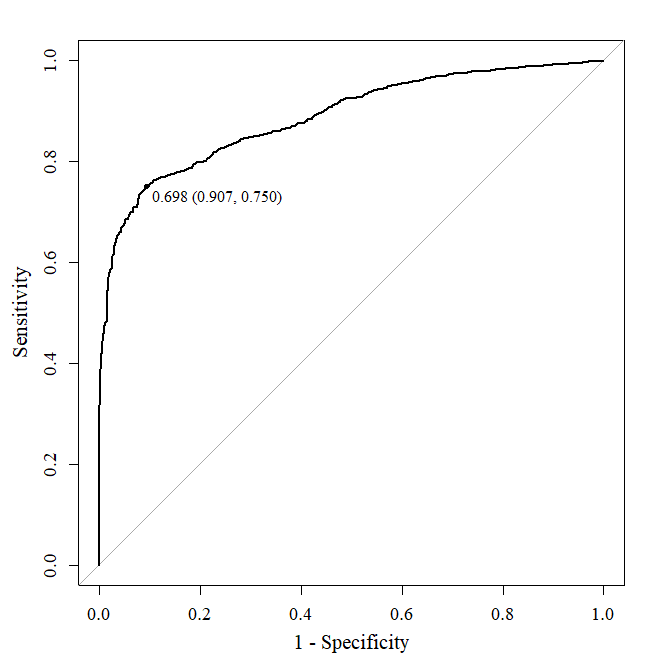 |

Fig. S2. Receiver operating characteristic curve for the confidence values

The numbers in the graph indicate the optimal cutoff values for confidence value, with the values in parentheses representing the corresponding “sensitivity, specificity.” The optimal cutoff values are calculated using Youden’s index
